# Identifying Phlorofucofuroeckol-A as a Dual Inhibitor of Amyloid-β_25-35_ Self-Aggregation and Insulin Glycation: Elucidation of the Molecular Mechanism of Action

**DOI:** 10.3390/md17110600

**Published:** 2019-10-23

**Authors:** Su Hui Seong, Pradeep Paudel, Hyun Ah Jung, Jae Sue Choi

**Affiliations:** 1 Department of Food and Life Science, Pukyong National University, Busan 48513, Korea; seongsuhui@naver.com (S.H.S.); phr.paudel@gmail.com (P.P.); 2 Department of Food Science and Human Nutrition, Jeonbuk National University, Jeonju 54896, Korea

**Keywords:** phlorotannin, amyloid-β aggregation, insulin glycation, dynamic simulation

## Abstract

Both amyloid-β (Aβ) and insulin are amyloidogenic peptides, and they play a critical role in Alzheimer’s disease (AD) and type-2 diabetes (T2D). Misfolded or aggregated Aβ and glycated insulin are commonly found in AD and T2D patients, respectively, and exhibit neurotoxicity and oxidative stress. The present study examined the anti-Aβ_25-35_ aggregation and anti-insulin glycation activities of five phlorotannins isolated from *Ecklonia stolonifera*. Thioflavin-T assay results suggest that eckol, dioxinodehydroeckol, dieckol, and phlorofucofuroeckol-A (PFFA) significantly inhibit Aβ_25-35_ self-assembly. Molecular docking and dynamic simulation analyses confirmed that these phlorotannins have a strong potential to interact with Aβ_25-35_ peptides and interrupt their self-assembly and conformational transformation, thereby inhibiting Aβ_25-35_ aggregation. In addition, PFFA dose-dependently inhibited d-ribose and d-glucose induced non-enzymatic insulin glycation. To understand the molecular mechanism for insulin glycation and its inhibition, we predicted the binding site of PFFA in insulin via computational analysis. Interestingly, PFFA strongly interacted with the Phe1 in insulin chain-B, and this interaction could block d-glucose access to the glycation site of insulin. Taken together, our novel findings suggest that phlorofucofuroeckol-A could be a new scaffold for AD treatment by inhibiting the formation of β-sheet rich structures in Aβ_25-35_ and advanced glycation end-products (AGEs) in insulin.

## 1. Introduction

The aberrant aggregation of misfolded proteins within a biological system is responsible for various pathological conditions. Protein aggregates commonly form and accumulate during normal aging, and it remains unclear whether misfolded proteins are a cause or consequence of aging [1]. In any case, protein aggregates are the major hallmarks of numerous neurodegenerative and metabolic disorders, and many central nervous system pathologies are associated with protein aggregation, such as amyloid-β peptide (Aβ) and tau protein aggregates in Alzheimer’s disease (AD), α-synuclein in Parkinson’s disease (PD), and the huntingtin protein in Huntington’s disease [2].

The Aβ peptide is a byproduct of proteolytic processing of the amyloid precursor protein, a transmembrane protein, by β- and γ-secretases. The initial cleavage of Aβ forms soluble and non-toxic monomers of varying lengths, the most common of which are the Aβ_1–40_ and Aβ_1–42_ peptides. 

However, when the monomers combine into clusters, they form Aβ oligomers which go on to form insoluble fibrils known as β-sheets [3]. That process is known as amyloid aggregation, and it eventually produces insoluble plaques. Aβ self-assembly is a complicated multi-phase process governed by noncovalent interactions with a delicate balance of entropic and enthalpic contributions [4]. Many physical factors can contribute to the development of strains and in how the β-sheets pack and the strands H-bond to each other: temperature, pH, concentration, ionic strength, agitation conditions such as sonication, and the presence of seeds [5]. Conventionally, Aβ fibrils have been considered the neurotoxic species primarily responsible for AD, but recent correlational research with the symptoms of dementia has found that soluble oligomers are the most cytotoxic Aβ forms [6]. A healthy body produces Aβ proteins at physiological concentrations that are essential for normal memory function and synaptic plasticity [7]. However, those same proteins at the high levels seen in AD are associated with synaptic dysfunction and memory loss [8]. Therefore, inhibiting amyloid aggregation or disaggregating pre-aggregated amyloid peptide is one therapeutic approach to treating AD. 

Insulin is a major hormone for regulating glucose homeostasis, and it can be non-enzymatically glycated by glucose and other reactive carbonyls under hyperglycemic conditions. Glycated insulin is less effective in controlling glucose homeostasis and stimulating glucose uptake than non-glycated insulin, and thus glycation might contribute to the insulin resistance and glucose intolerance of type 2 diabetes (T2D) [9]. Non-enzymatic protein glycation is an irreversible modification that begins with a chemical reaction between reducing sugars and primary amino groups, produces additional rearrangements to form a stable Amadori product, and eventually leads to the production of advanced glycation end-products (AGEs) [10]. An accumulation of AGEs is a feature of diabetic complications such as nephropathy [11], retinopathy [12], and atherosclerosis [13] and of neurodegenerative diseases such as AD. A receptor for advanced glycation end products (RAGE) is a multi-ligand transmembrane receptor expressed in several cell types that recognizes various ligands, including AGEs and Aβ. Cellular effects induced by protein glycation have been reported to be mediated by RAGE [10]. Previously, ribosylated insulin decreased cell viability and triggered death pathway through the activation of caspases-3 and -7, intracellular reactive oxygen species (ROS) production, and nuclear factor-κB (NF-κB) activation in NIH-3T3 mouse embryonic fibroblasts [10]. In addition, an AGEs–RAGE pathway triggers the pathogenesis of Aβ and tau hyperphosphorylation via activation of glycogen synthase kinase 3β and induces oxidative stress and neuro-inflammation via NF-κB activation [14]. Therefore, the inhibition of insulin glycation could be an important strategy for preventing AD, T2D, and diabetic complications.

Phlorotannins, which are polymers of phloroglucinol elements, are abundant in *Ecklonia* species of brown algae and have recently attracted much interest among neurodegeneration researchers. Due to the profound biological activities of marine-derived phlorotannins, including antioxidant and anti-inflammatory [15], antiviral [16], anti-cancer [17], anti-melanogenic [18], anti-adipogenic [19], and anti-diabetic [20] properties, research on their neuroprotective effects is emerging. Recently, we demonstrated that the molecular mechanism of the neuroprotective effects of eckol depended on monoamine oxidase-A inhibition and dopamine D_3_/D_4_ receptor agonism [21,22]. Similarly, dieckol and phlorofucofuroeckol-A (PFFA) showed MAOs-A/B inhibition, D_3_R/D_4_R receptor agonism, and D_1_/5HT_1A_/NK_1_ receptor antagonism [23]. In addition, we discovered that the anti-AD activity of phlorotannins, including eckol, dioxinodehydroeckol, dieckol, and PFFA, occurred via inhibition of β-secretase and acetylcholinesterase [24,25].

Previously, Kang et al. reported that *n*-butanol fraction obtained from *Ecklonia cava* significantly inhibited the oligomerization and fibrillation of Aβ_1-42_ [26]. Cho and coworkers reported that eckol and dieckol are abundant in the *n*-butanol fraction of *E. cava* ethanolic extract, with respective quantities of 37.55 and 115.0 mg/g [27]. However, no one has reported the effect of phlorotannins against Aβ self-aggregation. 

**Figure 1:**
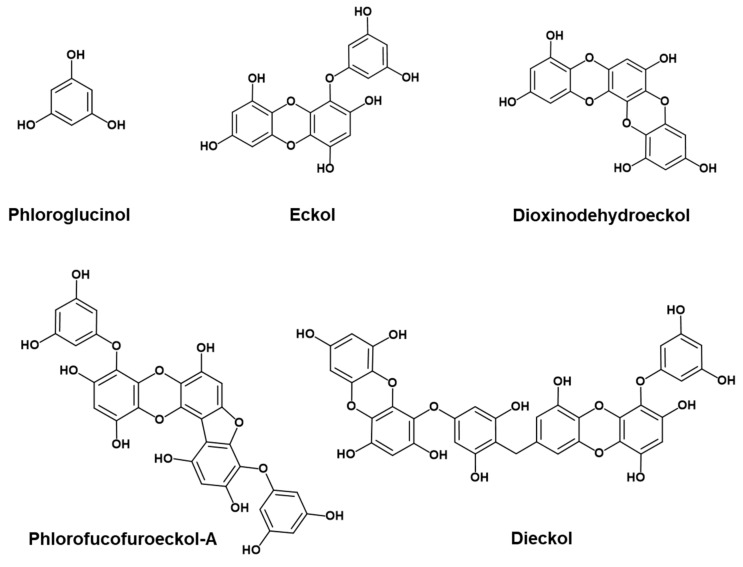
Structures of phlorotannins.

It is both nutritionally and pharmaceutically important if phlorotannins derived from edible brown seaweeds can inhibit Aβ aggregation and insulin glycation because those processes are closely related to the pathogenesis of AD. Therefore, our main aim in this study was to characterize the inhibitory effects of various phlorotannins (Figure 1) against self-induced Aβ_25-35_ aggregation and non-enzymatic insulin glycation and to provide molecular insights via molecular dynamics (MD) simulations of the inhibition of Aβ self-aggregation and insulin glycation. To the best of our knowledge, this study is the first to identify phlorotannins as dual inhibitors of both Aβ_25-35_ self-aggregation and insulin glycation.

## 2. Results

### 2.1. Inhibition of Aβ_25-35_ Self-Aggregation by Phlorotannins

**Figure 2:**
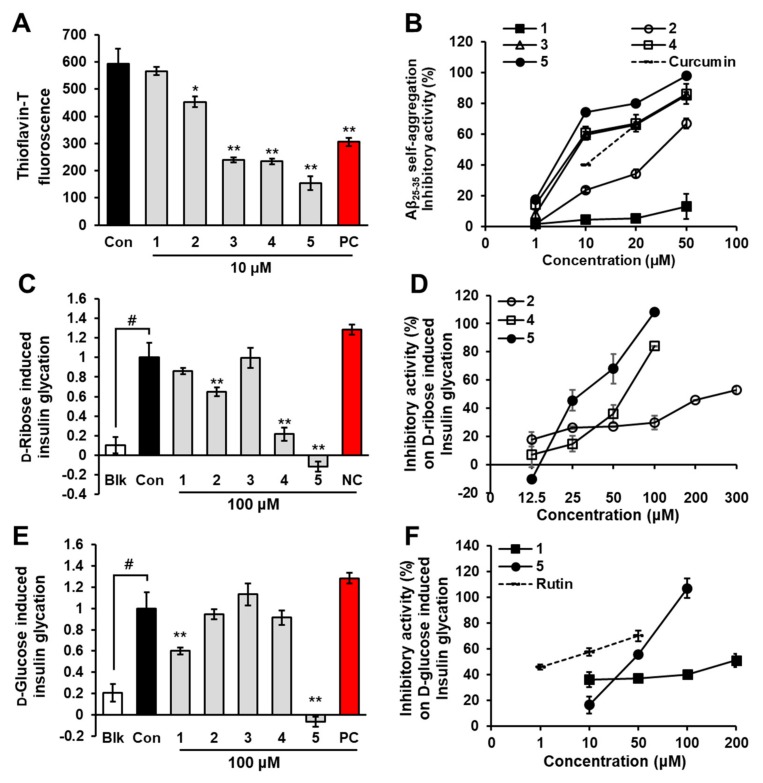
Effects of phloroglucinol (**1**), eckol (**2**), dioxinodehydroeckol (**3**), dieckol (**4**), and PFFA (**5**) on Aβ_25-35_ self-aggregation (**A**) and insulin glycation (**C** and **E**). Dose-dependent inhibitory activity of phlorotannins on Aβ_25-35_ self-aggregation (**B**) and insulin glycation (**D** and **F**). Values are expressed as mean ± SD (*n* = 3). ^#^ *p* < 0.01 indicates a significant difference from the blank group (Blk). * *p* < 0.05 and ** *p* < 0.001 indicate significant differences from the control group (Con). (Con: aggregated Aβ_25-35_ (100 μM) for A; glycated insulin group for C and E, **1**–**5**: Aβ_25-35_ + tested phlorotannins for A; insulin + d-ribose or d-glucose + tested phlorotannins for C and E, PC: curcumin for A; rutin for E, NC: vanillin).

We screened the inhibitory effects of five phlorotannins on Aβ_25-35_ self-aggregation at a concentration of 10 μM using thioflavin-T fluorescence. To verify our experiments, we used curcumin as a standard compound. As shown in Figure 2A, thioflavin-T fluorescence decreased significantly in the presence of eckol (*p* < 0.05), dioxinodehydroeckol (*p* < 0.001), dieckol (*p* < 0.001), and PFFA (*p* < 0.001) at 10 μM. Among them, PFFA showed the strongest inhibitory effect with 80.00% ± 5.5% inhibition, followed by dieckol, dioxinodehydroeckol, and eckol with inhibitions of 66.98% ± 1.5%, 66.07% ± 2.5%, and 34.45% ± 1.5%, respectively. However, phloroglucinol showed no inhibitory effect on Aβ_25-35_ self-aggregation even at 50 μM. As shown in Figure 2B, eckol, dioxinodehydroeckol, dieckol, and PFFA had dose-dependent inhibitory effects on Aβ_25-35_ self-aggregation. We obtained the 50% inhibitory concentration (IC_50_) of phlorotannins for Aβ_25-35_ self-aggregation from the dose-activity graph and found it to be in the range of 6.18 to 34.36 μM (Table 1). Notably, PFFA, dieckol, and dioxinodehydroeckol exhibited lower IC_50_ values (6.18 ± 0.18, 7.93 ± 0.16, and 8.31 ± 0.23 μM, respectively) than the standard compound, curcumin (10.73 ± 1.40 μM).

### 2.2. Inhibition of Insulin Glycation by Phlorotannins

Glycated bovine insulin was observed by fluorescence spectroscopy because AGEs are marked by a typical fluorescence emission at 410 nm (excitation at 320 nm). To verify our experimental condition, we used vanillin as a negative control for d-ribose-induced protein glycation [28] and rutin as a positive control for d-glucose-induced protein glycation [29].

As shown in Figure 2C, fluorescence intensity after a 1-week incubation of bovine insulin and d-ribose increased significantly compared to the blank group (*p* < 0.001). However, in the presence of 100 µM eckol, PFFA, or dieckol, a significant reduction of insulin glycation was detected, as indicated by a decline in fluorescence intensity. Those inhibitory activities of eckol, PFFA, and dieckol were dose-dependent, with IC_50_ values of 258.54 ± 10.81, 29.50 ± 0.53, and 63.67 ± 3.83 µM, respectively (Figure 2D). Phloroglucinol and dioxinodehydroeckol showed weak or no inhibitory activity on d-ribose-induced insulin glycation at 100 µM, and the negative control (vanillin) showed no activity at 500 µM. 

Similarly, fluorescence intensity after a 2-weeks incubation of insulin and d-glucose increased remarkably compared to the blank group (Figure 2E). In the presence of PFFA, fluorescence intensity was dose-dependently reduced with an IC_50_ value of 43.55 ± 2.38 µM. In addition, phloroglucinol showed 40.02% inhibition at 100 µM. However, other phlorotannins showed no inhibitory activity on d-glucose-induced insulin glycation under the tested concentration. 

### 2.3. Prevention of Lipid Peroxidation in Whole Rat Brain Homogenates by Phlorotannins

**Figure 3:**
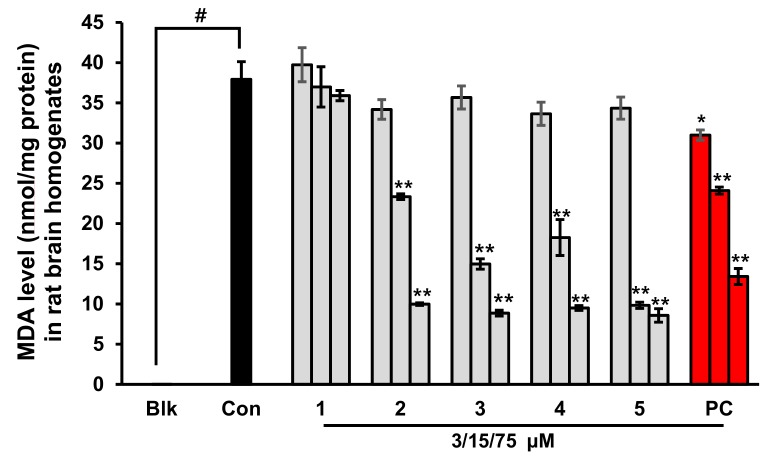
Effects of phloroglucinol (**1**), eckol (**2**), dioxinodehydroeckol (**3**), dieckol (**4**), and PFFA (**5**) on lipid peroxidation in whole rat brain homogenates. Values are expressed as mean ± SD (*n* = 3). * *p* < 0.01 and ** *p* < 0.001 indicate significant differences from the Con group (Blk: without Fe^2+^ or TBA, Con: with Fe^2+^ and TBA, **1**–**5**: with Fe^2+^, TBA, and the tested phlorotannins, PC: Trolox).

A combination of H_2_O_2_ and Fe^2+^ was used to initiate lipid peroxidation in the brain via Fenton’s reaction [30]. We evaluated the inhibitory activity of phlorotannins on lipid peroxidation using Fe^2+^-treated rat brain homogenates. In the control group, the malondialdehyde (MDA) level was significantly elevated compared with the blank group (not treated with Fe^2+^ or TBA; *p* < 0.001), as shown in Figure 3. However, the MDA level was significantly decreased in the presence of eckol, dioxinodehydroeckol, dieckol, PFFA, or Trolox (positive control). Among the tested phlorotannins, PFFA best prevented lipid peroxidation, with an EC_50_ value of 10.96 ± 0.16 µM, followed by dioxinodehydroeckol, dieckol, and eckol with respective EC_50_ values of 12.43 ± 1.50, 13.51 ± 0.38, and 38.64 ± 1.16 µM (Table 1). Interestingly, PFFA, dioxinodehydroeckol, and dieckol exhibited lower EC_50_ values than the positive control, Trolox (EC_50_ = 49.01 ± 3.50 µM).

### 2.4. Docking Simulation for Phlorotannins on Aβ_25-35_

**Figure 4:**
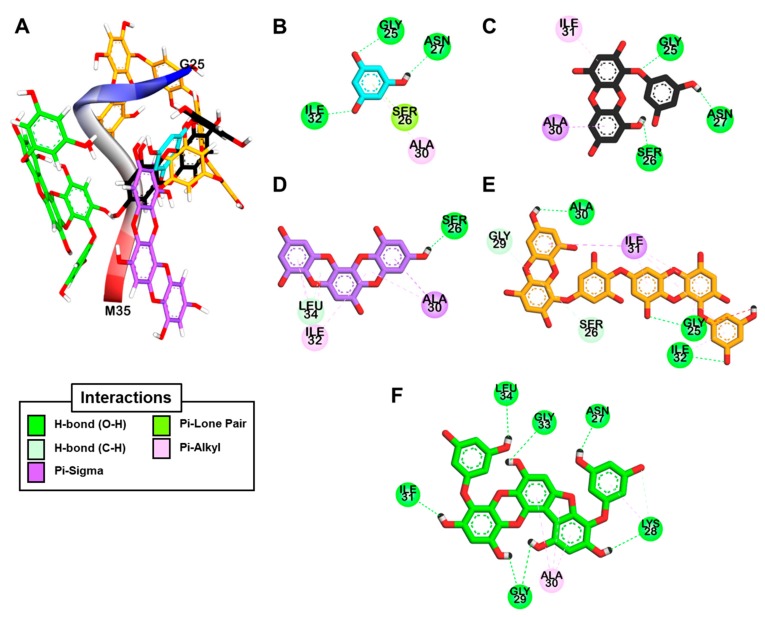
The best docked poses of phloroglucinol (cyan stick), eckol (black stick), dioxinodehydroeckol (purple stick), dieckol (orange stick), and PFFA (green stick) bound to the Aβ_25-35_ peptide (**A**) and a detailed 2D view of the phlorotannin–peptide interactions (**B**–**F**).

We conducted an in silico docking analysis to find the most stable binding site of the phlorotannins on Aβ_25-35_ (sequence GSNKGAIIGLM). The predicted binding site residues and maximum binding affinities for the phlorotannins are presented in Table 2. The five phlorotannins showed a negative binding energy to Aβ_25-35_ and docked near it. As shown in Figure 4e,f, dieckol and PFFA formed 5- and 7-hydrogen bonding interactions with Aβ_25-35_, respectively, along with pi-interactions between the aromatic rings of these compounds and hydrophobic residues such as Ala30–Ile31–Ile32. The aromatic ring of dioxinodehydroeckol (Figure 4D) formed many pi-interactions with Ala30 and Ile32. As shown in Figure 4C, the OH group of eckol formed three hydrogen bonds with the Gly25‒Asn27 residues, and the aromatic ring of eckol interacted with Ile31 and Ala30 via pi-interactions. These results indicate that bulky compounds of more than three repeating phloroglucinol units interacted evenly with most Aβ_25-35_ residues. However, the phloroglucinol monomer is not expected to cause structural changes to Aβ_25-35_ because of its very small structure, although it did form hydrogen bonds with the Gly25, Asn27, and Ile32 residues of the peptide, as shown in Figure 4B. On the other hand, dioxinodehydroeckol and PFFA, which have two dibenzo-1,4-dioxin or dibenzofuran linkages, retain fewer rotatable bonds than the other phlorotannins, so they showed low torsion energies and stably interacted with the Aβ_25-35_ with a high binding affinity.

### 2.5. Dynamic Simulation of Phlorotannins Inhibiting Aβ_25-35_ Self-Aggregation

**Figure 5:**
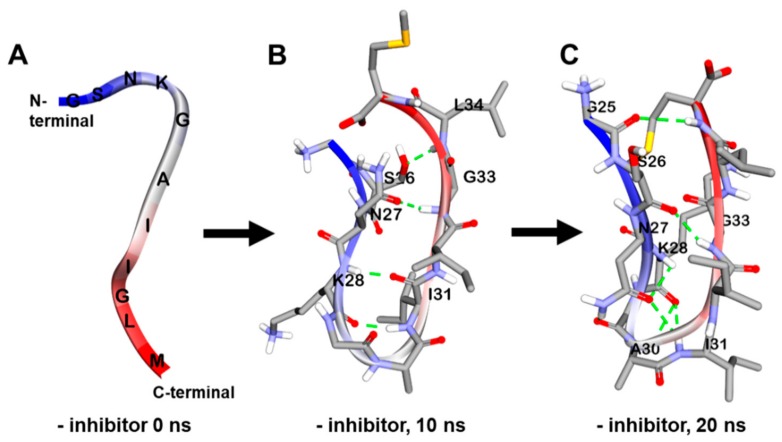
Molecular dynamics (MD) simulation trajectories of Aβ_25-35_ peptide at times *t* = 0 (**A**), 10 (**B**), and 20 ns (**C**).

**Figure 6:**
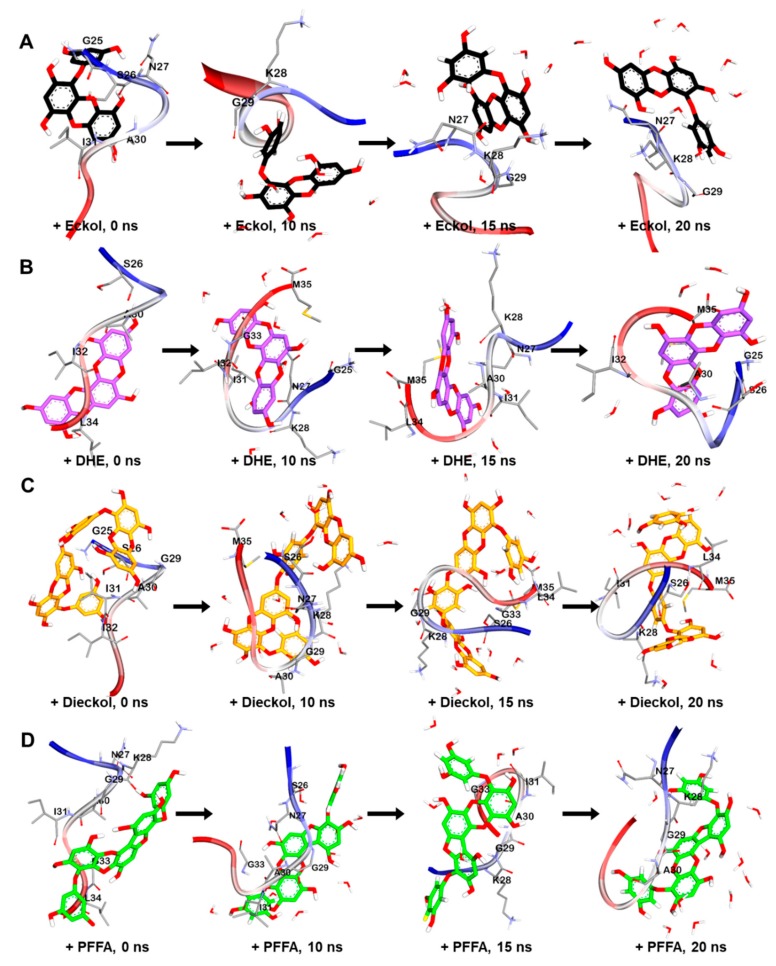
MD simulation trajectories of eckol (**A**), dioxinodehydroeckol (DHE, **B**), dieckol (**C**), and phlorofucofuroeckol-A (PFFA, **D**) bound to Aβ_25-35_ peptide at times *t* = 0, 10, 15, and 20 ns.

In the absence of inhibitors, the amorphous form of Aβ_25-35_ changed into the β-sheet form with many internal hydrogen bonds between strands in a 150 mM NaCl aqueous solution during a 20 ns MD simulation (Figure 5A–C). We analyzed and visualized the evolution of the secondary structure during that 20 ns MD simulation using VMD. As shown in Figure 6A, the β-sheet began being generated at 5.9 ns and continued forming until 20 ns. 

To understand the binding modes between Aβ and phlorotannins, we subjected the most stable phlorotannin–Aβ_25-35_ complexes from the docking study to MD simulation.

**Figure 7:**
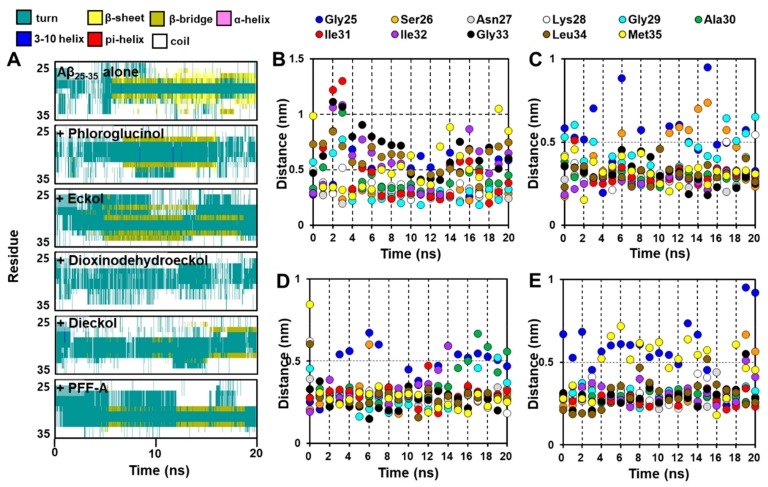
Evolution of secondary structures with time (ns) for Aβ_25-35_ peptide with and without phlorotannins (**A**). Minimum distances (ns) between the residues in the Aβ_25-35_ peptide and the phlorotannins (**B** for eckol, **C** for dioxinodehydroeckol, **D** for dieckol, and **E** for PFFA) during 20 ns MD simulations.

As shown in Figure 7A, the MD simulation results suggest that eckol interacts favorably with the Asn27–Lys28–Gly29 residues of the peptide. After 20 ns, the hydroxyl moiety of the eckol formed strong hydrogen bonds with the Asn27 and Gly29 residues, and the aromatic ring of this compound interacted with Lys28 via an amide-pi stacked bond. Those interactions between the peptide and eckol increased the β-bridge content during the 20 ns MD simulation. However, unlike Aβ25-35 alone, no β-sheet was observed in the presence of eckol (Figure 6A).

Dioxinodehydroeckol formed many interactions with most of the peptide residues, which eliminated all β-structures, including β-sheet and β-bridge formations (Figure 6A). Although it did not interact with Met35 and Ile31, which are assumed to play a key role in the assembly and neurotoxicity of Aβ [31], in the best docked pose (Figure 4D), dioxinodehydroeckol continuously interacted with those residues via hydrophobic and hydrophilic bonds during the MD simulations. After 20 ns of simulation, dioxinodehydroeckol formed seven hydrogen bonds with the Ser26, Ile32, Gly25, and Met35 residues. In addition, the aromatic ring of this compound interacted with Ala30 and Met35 via several pi-interactions: pi-sigma, pi-sulfur, and pi-alkyl bonds (Figure 7B).

As shown in Figure 7C, dieckol strongly interacted with Met35 and Lys28 via hydrogen bonds during the simulations. In addition to Met35, dieckol continuously reacted with the Ser26, Lys28, Ile31, and Leu34 residues. After the 20 ns MD simulation, hetero oxygen atoms and the hydroxyl moiety of dieckol formed hydrogen bonds with Met35 and Lys28. Pi-interactions were also detected between the aromatic rings of dieckol and the Met35, Ile31, Leu34, Ser26, and Lys28 residues.

**Figure 8:**
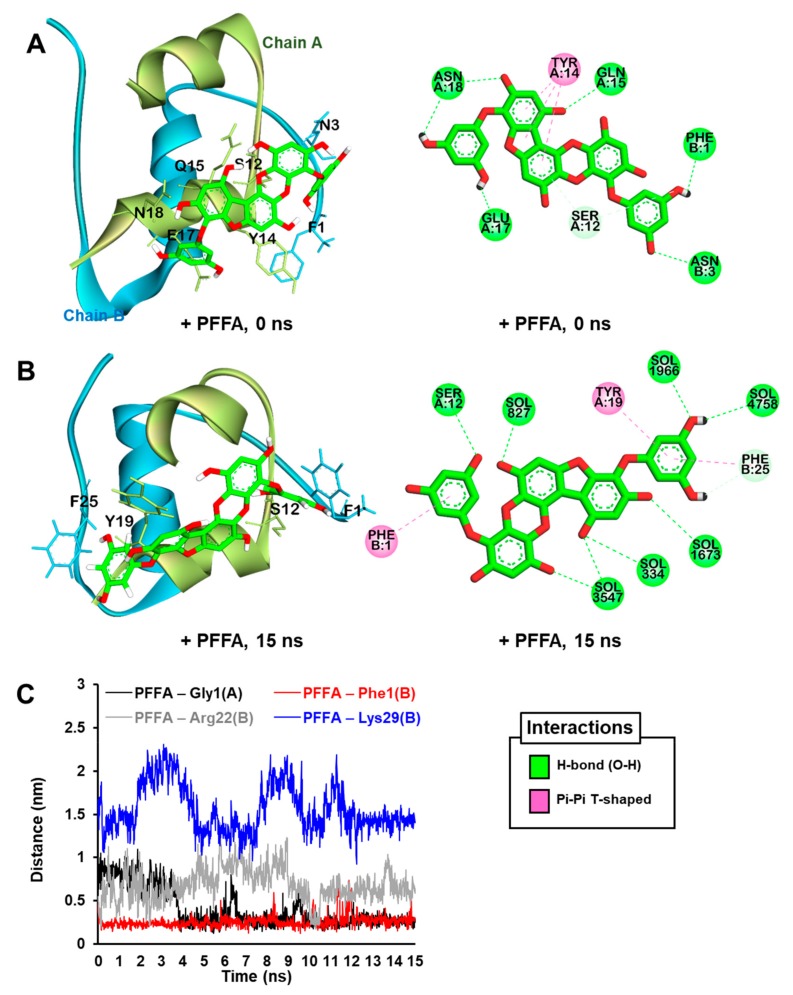
The best predicted pose from the molecular docking simulation for PFFA (green stick) binding to bovine insulin (**A**). The MD trajectories for PFFA bound to bovine insulin at 15 ns of the MD simulation (**B**). Water molecules are not shown in the 3D view for clarity. Hydrogen bonds, pi-donor hydrogen bonds, and pi-amide stacked interactions are shown as green, pale green, and pink dashed lines, respectively. Water molecules are labeled “SOL” in the 2D view. Minimum distances (nm) between the glycation site residues in the bovine insulin and PFFA during 15 ns MD simulations (**C**).

PFFA (Figure 7D) interacted favorably with the Ile31, Ala30, Gly29, and Gly33 residues during the simulations and effectively interrupted the self-assembly of Aβ_25-35_. After the 20 ns simulation, this compound formed a hydrogen bond with Asn27 and a strong electrostatic interaction (pi-cation) with Lys28. In addition, hydrophobic interactions were observed between PFFA and the Gly29 and Lys28 residues. Furthermore, a secondary structure analysis revealed that the formation of a β-sheet decreased significantly in the presence of dieckol and PFFA, as shown in Figure 8A. 

We also analyzed the minimum distances between each residue in the peptide and the phlorotannins during the 20 ns simulations. As shown in Figure 6B, dieckol, PFFA, and dioxinodehydroeckol moved to within 0.5 nm of the Aβ_25-35_ during the simulations, whereas eckol moved to within 1.0 nm of the peptide. In addition, these four phlorotannins continuously interacted with Asn27 and Ile31 at a close distance during the 20 ns MD simulations. Dioxinodehydroeckol and dieckol interacted closely with Gly33, Leu34, and Met35. PFFA did not interact closely with Met, but it did compactly interact with other hydrophobic residues, including Ile31-Ile32-Gly33-Leu34. However, phloroglucinol could not access the Aβ_25-35_ peptide until 3 ns into the 20 ns MD simulation even though the simulation began with a docked phloroglucinol–Aβ_25-35_ complex (data not shown).

### 2.6. Docking Simulation for PFFA on Bovine Insulin

The most stable binding site of PFFA on the bovine insulin was analyzed using an in silico automated docking study. PFFA showed a negative binding energy (−5.03 kcal/mol) to the bovine insulin. As shown in Figure 8A and Table 2, the hydroxyl moieties of the PFFA interacted with Phe1 and Asn3 in chain B and Ser12, Gln15, Glu17, and Asn18 in chain A of the insulin via hydrogen bonds. In addition, the dibenzofuran ring of the PFFA interacted with Tyr14 in chain A via a pi-amide stacked interaction.

### 2.7. Dynamic Simulation of PFFA on Bovine Insulin

To understand the binding modes between insulin and PFFA, we subjected the most stable PFFA–insulin complex (Figure 8A) obtained from the docking study to MD simulation. 

As shown in Figure 8B, the MD results reveal that PFFA moved slightly closer to Phe25 (chain B) over time, and the PFFA-insulin complex was finally stabilized by intra-interactions between PFFA and insulin residues, including Ser12 and Tyr19 in chain A and Phe1 and Phe25 in chain B.

Furthermore, minimum distances (nm) between PFFA and major glycation site residues including Phe1, Arg22, and Lys29 in chain B and Gly1 in chain A of insulin over MD run times are described in Figure 8C. PFFA formed stable interaction with Phe1 in chain B over the entire runs, whereas PFFA interacted with Gly1 in chain A via H-bond from 5 ns until 10 ns runs (data not shown). In addition, PFFA weakly interacted with Arg22 and could not reach near Lys29 residue during 15 ns MD runs.

## 3. Discussion

Phlorotannins, a natural polyphenol found abundantly in brown seaweeds (especially in the *Ecklonia* species), are known to have a diverse range of pharmacological activity. As neuroscience research progresses, the many neuroprotective effects of various phlorotannins are being reported, including inhibitory activity against enzymes linked to the pathogenesis of AD and PD [22,24,25,32], modulatory activity against G-protein coupled receptors related to neuronal diseases such as PD and psychological diseases [22,23], and free-radical scavenging activity [15]. Although some reports have indicated that phlorotannins have protective effects against the neurotoxicity of the Aβ_1-42_ oligomer [32] or Aβ_25-35_ peptides [33,34] in neuronal cell-lines, phlorotannins have not previously been studied as an inhibitor of Aβ self-aggregation.

Soluble Aβ oligomers and insoluble fibrils are known to bind several receptors at the cell surface, including the insulin receptor (IR), RAGE, α-7-nicotinic acetylcholine receptor, β2 adrenergic receptor, N-methyl-d-aspartic acid receptor, and toll-like receptor 2 [35]. Many of the signaling pathways initiated by those receptors converge into common downstream targets that are ultimately responsible for synaptic impairment, neurotoxicity, and cell death. The predominant forms of the Aβ present in aggregates are Aβ_1–40_ and Aβ_1–42_. Aggregates of shorter fragments such as Aβ_25–35_ result from the cleavage of soluble racemized Aβ_1-40_ peptides and are observed in the brain tissue of AD patients [36]. The Aβ_25–35_ fragment is the smallest that retains both the toxicity and aggregation properties of the full-length molecule [37]. In a previous study, Aβ_25–35_ showed outstanding speed of aggregation and immediate cytotoxicity in vitro compared with other forms [38]. The Aβ_25–35_ treated animals exhibit a statistically relevant cognitive impairment as well as alteration of the key markers of cell death and neurodegeneration [39]. Therefore, to evaluate whether phlorotannins could prevent the self-aggregation of Aβ, we tested their potency using Aβ_25–35_ as the target.

As with previous reports [38], the thioflavin T fluorescence of Aβ_25–35_ increased after 24 h’ incubation compared with freshly prepared Aβ_25–35_. However, the thioflavin T intensity decreased significantly when Aβ_25–35_ was co-incubated with eckol, dioxinodehydroeckol, dieckol, or PFFA, though phloroglucinol showed no effect at the tested concentrations. Among them, PFFA (a pentamer of a phloroglucinol unit with both dibenzo-1,4-dioxin and dibenzofuran linkages), dieckol, and dioxinodehydroeckol (a trimer of phloroglucinol with two dibenzo-1,4-dioxin linkages) showed strong potency, with an IC_50_ range of 6.18 to 9.06 μM. Eckol, a trimer of phloroglucinol with a dibenzo-1,4-dioxin linkage, showed less activity than the others. These outcomes imply that having both dibenzo-1,4-dioxin and dibenzofuran linkages (or two dibenzo-1,4-dioxin linkages) in the phlorotannin scaffolds are essential to effectively prevent the self-assembly of Aβ_25–35_. 

Our molecular docking and MD simulation studies have clearly demonstrated the binding modes between the peptide and phlorotannins. After a 20 ns MD simulation, Aβ_25–35_ alone showed β-sheet content and had β-turn content at Gly29-Ala30, in accord with the results of a previous study [40]. However, when bound to eckol, dioxinodehydroeckol, dieckol, or PFFA, the peptide had significantly less β-sheet content and existed in an amorphous state. The MD analysis revealed that these four phlorotannins commonly interacted with the Asn27 and Ile31 residues, though their interactions differed. Eckol and dioxinodehydroeckol favorably interacted with hydrophilic residues, including Asn27-Lys28-Gly29, whereas dieckol and PFFA mainly interacted with the C-terminus hydrophobic residues, including Ile31-Ile32-Gly33-Leu34-Met35. It was previously reported that Aβ_25–35_ assemblies are mediated by side-chain to side-chain hydrogen interactions in the Asn27-Ile32 region. In addition, an experimental analysis conducted by Pike and coworkers confirmed that the Leu34-Met35 region is essential to Aβ aggregation, and Met35 is important for the neurotoxicity of Aβ_25–35_/_1-40_ [31,38]. Therefore, the different binding aspects of the phlorotannins could explain their different potency against Aβ_25–35_ aggregations in vitro. However, it was reported that GROMOS force fields showed strongly biased results toward β-sheet structures [41] and the drug-binding sites of Aβ monomers and small oligomers are very transient [42], thus further extensive MD simulation using other CHARMM, AMBER99-ILDN, or AMBER14SB force fields, which showed good balanced results in structures as well as kinetics, should be conducted to confirm our MD results [42,43].

For many years, it was commonly believed that the brain was insensitive to insulin. However, it is now acknowledged that insulin has vital neuro-modulatory functions, such as the regulation of glucose homeostasis and roles in cognition, learning, and memory, which are impaired in AD [44]. In addition, insulin can prevent the formation of the Aβ_1-42_ oligomer and ameliorate the Aβ_1-42_-induced impairment of long-term potentiation in hippocampal slices [45]. But once insulin is glycated under hyperglycemic or diabetic conditions, it cannot bind IR or block Aβ aggregation, which produces a decline in IR-mediated signaling pathways and can facilitate Aβ-mediated brain damage [9,45]. Glucose is the most abundant reducing sugar in vivo with its plasma concentrations ranging from 70 to 140 mg/dL in healthy individuals, while two times higher in T2D patients [46]. In addition, abnormally high doses of d-ribose have been found in the urine of T2D patients [47], and ribosylated insulin was found to exhibit significant cytotoxicity in NIH-3T3 cells [10].

Although some reports have suggested that natural products, such as vanillin, rutin, quercetin, and pinocembrin, can act as insulin glycation inhibitors [28], studies about insulin glycation remain inadequate. Therefore, in this study, we evaluated the inhibitory effects of phlorotannins on d-glucose or d-ribose-induced non-enzymatic and irreversible glycation of bovine insulin, and we elucidated the molecular mechanism of action for insulin glycation and its inhibition by phlorotannins.

PFFA and dieckol showed remarkably potent inhibition of d-ribose induced insulin glycation at less than 100 µM. However, other phlorotannins showed weak or no activity at the tested concentrations. In the case of d-glucose induced insulin glycation assay, only PFFA showed significant inhibitory activity at 100 µM. Our results suggest that PFFA might be promising lead compounds against non-enzymatically glycated insulin–mediated pathogenesis. 

Glucose-binding sites (Phe1, Val2, Leu17, Arg22, and Lys29 in chain B; Glu1 in chain A) on insulin had already been elucidated via in silico prediction and mass spectrometry studies [48,49]. Our docking and MD simulation analyses clearly show the binding modes between bovine insulin and PFFA. Phe1 in chain B of insulin, which is the major glycation site of insulin, connected strongly with PFFA via H-bond and pi-pi bonds during MD runs. Our computational prediction also indicated that PFFA could interact with Ser12, Tyr19, and Phe25 residues, which could inhibit insulin glycation by disturbing the interaction between glycation site of insulin and d-glucose (or d-ribose).

In the brain, Aβ plaques and AGEs can be major sources of oxidative stress [14,37]. Brains are highly susceptible to oxidative damage because their membranes contain high amounts of polyunsaturated and peroxidable fatty acids and have a high rate of oxygen consumption [50]. In our study, eckol, dioxinodehydroeckol, dieckol, and PFFA dose-dependently reduced MDA levels in whole rat brain tissue homogenate. Dioxinodehydroeckol, dieckol, and PFFA showed strong activity, and eckol had moderate potency. Thus, phlorotannins with more than three repeating phloroglucinol units are required to prevent lipid peroxidation. However, in the homogenates, destroying the structures and cells via a myriad of processes that is started cannot occur in the living organism and almost all unspecific antioxidants could inhibit this nonspecific peroxidation [51,52]. Therefore, further mechanism studies are required to confirm this activity. 

AD drugs are required to enter the blood-brain barrier (BBB) to achieve therapeutic levels in the central nervous system (CNS). Pharmacokinetic parameter prediction study indicated that eckol penetrates the CNS moderately [22]. In addition, Kwak et al. reported that dieckol, with a number of hydroxyl groups and high molecular weight, effectively passed the BBB in rats upon intravenous injection [53]. Studies of PFFA in BBB permeability has not been implemented, but properties similar to dieckol may be anticipated. To strengthen the penetration property of BBB, several methods were developed using nanoparticles, aromatic substances (e.g., borneol), and chemical drug delivery systems [54]. Recently, Venkatesan et al. successfully biosynthesized the silver nanoparticles using *E. cava* [55]. Therefore, we are able to overcome the limitation of phlorotannins over BBB penetration. 

We are the first to identify the inhibitory activity of phlorotannins as dual Aβ aggregation and insulin glycation inhibitors, and our computational study clearly shows the mechanism by which PFFA inhibits Aβ self-aggregation and bovine insulin glycation. However, further in vivo experiments are needed to verify this in vitro and in silico prediction.

In conclusion, our results show that dieckol and PFFA derived from marine brown algae strongly reduce Aβ_25–35_ self-aggregation and non-enzymatic insulin glycation. In addition, we used docking and MD simulation studies to demonstrate the molecular mechanism by which the active phlorotannins inhibit Aβ aggregation and insulin glycation. Therefore, those phlorotannins can prevent neuronal damage by inhibiting the formation of β-sheet rich amyloid peptide structures and insulin glycation as well as by preventing lipid peroxidation, producing normal insulin and Aβ processing pathways. Taken together, our findings suggest that phlorotannins could be a promising therapeutic lead compound for the treatment of AD and T2D.

## 4. Materials and Methods 

### 4.1. Chemicals and Reagents

Amyloid β-protein fragment 25–35 (Aβ_25-35_), 1,1,1,3,3,3-hexafluoro-2-propanol, 1,1,3,3-tetramethoxypropane, Trolox, curcumin, d-ribose, d-glucose, vanillin, and insulin from bovine pancreas were purchased from Sigma-Aldrich (St. Louis, MO, USA). Sodium dodecyl sulfate (SDS) and thiobarbituric acid (TBA) were purchased from Biosesang (Seoul, Republic of Korea) and Tokyo Chemical Industry (Tokyo, Japan), respectively. All chemicals and solvents for column chromatography were of reagent grade from commercial sources and were used as received. 

### 4.2. Preparation of Phlorotannins

Five phlorotannins—phloroglucinol, eckol, dioxinodehydroeckol, dieckol, and PFFA—were isolated from the ethyl acetate fraction of *E. stolonifera* ethanolic extract as described by Yoon et al. [23]. The cemical structures of the isolaed phlorotannins are shown in Figure 1. 

### 4.3. Assay for Aβ_25-35_ Self-Aggregation

A monomeric Aβ_25-35_ solution was prepared using the method of Naldi and coworkers [56]. A 2.5 μL aliquot of various concentrations of the tested phlorotannins in 50 mM phosphate buffer (pH 7.4) with 100 mM NaCl was added to 72.5 μL of Aβ_25-35_ sample (100 μM), and the mixture was incubated at 4 °C for 1 day. After incubation, 25 μM thioflavin T in 50 mM glycine-NaOH buffer (pH 8.5) was added to the reaction mixture. Fluorescence emission intensity was monitored at 490 nm (λ_exc_ = 446 nm) using a fluorescence microplate reader (Gemini XPS, Molecular Devices, Sunnyvale, CA, USA). Curcumin was used as a standard compound.

### 4.4. Assay for Non-Enzymatic Insulin Glycation

The insulin from bovine pancreas was dissolved in third grade distilled water to a concentration of 6 mg/mL and acidified to pH 2.4 using phosphoric acid to produce monomeric insulin. Then the insulin solution was neutralized to pH 7.0 using 10 M NaOH. Non-enzymatic glycation of insulin was initiated by mixing the insulin solution, 0.5 M d-ribose (or 1.5 M d-glucose) in 50 mM NaH_2_PO_4_ buffer (pH 7.0), and 10% dimethyl sulfoxide (DMSO) or the test phlorotannins in a 1:8:1 ratio and incubating the mixture at 37 °C for 6 days (2 weeks for d-glucose induced insulin glycation). Vanillin was used as a negative control for d-ribose induced insulin glycation [27], whereas rutin was used as a positive control for d-glucose induced insulin glycation [28]. After incubation, the reaction mixture was measured at an excitation wavelength of 320 nm and emission wavelength of 410 nm using a fluorescence microplate reader (Gemini XPS).

### 4.5. Preparation of Rat Brain Homogenates

Whole rat brain homogenates were prepared from freshly killed Sprague Dawley rats (male, 6-months old) and provided by the Aging Tissue Bank of Pusan National University. One g of whole rat brain was homogenized in 10 mL of cold 20 mM sodium phosphate buffer containing 140 mM KCl (pH 7.4) and centrifuged at 1300× *g* for 15 min. 

### 4.6. Lipid Peroxidation Assay

A TBA reactive species (TBARS) assay was used to evaluate the antioxidant activity of the phlorotannins in the rat brain homogenates [57]. An aliquot of the supernatant fraction of the homogenates was mixed with phlorotannins dissolved in 10% DMSO, freshly prepared ferric sulfate (250 μM), and distilled water in a 10:5:3:12 ratio, and then the reaction mixture was incubated at 37 °C for 1 h. The reaction was completed by adding a color reagent containing 0.8% TBA, 8.1% SDS, and a 7.5% (final concentration) acetic acid-NaOH solution (pH 3.4) in a 2:1:2 ratios. The mixture was boiled in a water bath for 1 h. After cooling, the reaction mixture was mixed with an equal volume of *n*-butanol and centrifuged at 1300× *g* for 10 min. The absorbance of the upper layer was measured at 532 nm using a spectrophotometer (Molecular Devices). The formation of TBARS was expressed as MDA nmol/mg protein using 1,1,3,3-tetramethoxypropane as a standard. Trolox was used as a positive control.

### 4.7. Molecular Docking Simulation

Docking simulations were carried out with AutoDock 4.2 [58]. The X-ray crystallographic structures of Aβ_25-35_ and bovine insulin were obtained from the RCSB Protein Data Bank using codes 1QXC (model 1) [59] and 2ZP6, respectively. The chemical structures of the phlorotannins (phloroglucinol, eckol, eckstolonol, dieckol, and PFFA) were obtained from the PubChem database using codes 359, 145937, 10429214, 3008868, and 130976, respectively. The rotatable bonds were set by the Autodock tools, and all torsions were allowed to rotate during ligand preparation. Grid map files were generated using the Autogrid. For each phlorotannin-Aβ_25-35_ or PFFA-insulin complex, 10 docking poses were generated using the default genetic algorithm (GA) parameters. Further, Lamarckian GA was used to compute ligand conformations. The pose with the lowest binding energy was selected as the final docking result. Docking results were visualized using Discovery Studio (v17.2, Accelrys, San Diego, CA, USA).

### 4.8. Molecular Dynamic simulation

All simulations in this study were done using the open source GROMACS 2018.1 package [60] with a force field of GROMOS96 43A1 [61]. The most stable phlorotannin-Aβ_25-35_ and PFFA-insulin complexes obtained from the automated docking simulation were subjected to MD simulation. The type of N- (Gly25 for Aβ_25-35_; Gly1 and Phe1 for insulin) and C-terminals (Met35 for Aβ_25-35_; Asn21 and Ala30 for insulin) of the peptides were set as NH^3+^ and COO^–^, respectively. Each phlorotannin-peptide complex was placed in a single cubic box and solvated using the spc216 water model. The system was neutralized with appropriate numbers of Na^+^ and Cl^–^ ions using a 0.15 mM ion concentration (without an ion concentration for the PFFA-insulin complex). After that, the system was subjected to energy minimization by the steepest descent method with a maximum of 5000 steps and was optimized with the LINCS algorithm using 100 ps under constant volume and pressure [62]. Electrostatic interactions were calculated by the particle mesh Ewald process [63]. The pressure (1 bar) and temperature (300 K) of the system were controlled by the Parrinello–Rahman and V-rescale methods, respectively. The simulation results were scrutinized and visualized using Discovery Studio (v17.2, Accelrys, San Diego, CA, USA). The timeline for the secondary structure of Aβ_25-35_ peptide was investigated using visual molecular dynamics (VMD) [64].

### 4.9. Statistical Analysis

The 50% inhibitory concentration (IC_50_) values (μM) obtained from the dose-inhibition curves are expressed as the mean ± SD (*n* = 3). The Student’s *t*-test (two-tailed) was used to determine the significant differences between the blank and control or the phlorotannin-treated groups and control in Figure 2 and Figure 3.

## Figures and Tables

**Table 1:** Effect of phlorotannins on Aβ_25-35_ self-aggregation, bovine insulin glycation, and lipid peroxidation in rat brain homogenates.

Compounds	IC_50_ (μM) ^a^			EC_50_ (μM) ^a^
	Aβ_25-35_ Aggregation	d-Ribose-Induced Insulin Glycation	d-Glucose-Induced Insulin Glycation	Lipid Peroxidation
Phloroglucinol	>100	>100	>100	>75
Eckol	34.36 ± 1.11	258.54 ± 10.81	>100	38.64 ± 1.16
Dioxinodehydroeckol	8.31 ± 0.23	>100	>100	12.43 ± 1.50
Dieckol	7.93 ± 0.16	63.67 ± 3.83	>100	15.48 ± 2.14
Phlorofucofuroeckol-A	6.18 ± 0.18	29.50 ± 0.53	43.55 ± 2.38	10.96 ± 0.16
Curcumin ^b^	10.73 ± 1.40	‒	‒	‒
Vanillin ^c^	‒	>500	‒	‒
Rutin ^b^		‒	5.19 ± 1.35	
Trolox ^b^	‒	‒	‒	49.01 ± 3.50

^a^ The 50% inhibition concentration (IC_50_) and 50% effective concentrations (EC_50_) are expressed as mean ± SD, *n* = 3. ^b^ Curcumin, rutin, and Trolox were used as a positive control for the Aβ_25-35_ aggregation, d-glucose-induced insulin glycation and lipid peroxidation assays, respectively. ^c^ Negative control for the d-ribose-induced insulin glycation assay.

**Table 2:** Binding affinity and interacting residues of phlorotannins with human Aβ_25-35_ and bovine insulin peptides from docking analysis.

Ligands	Binding Energy (kcal/mol)	Hydrogen Bonding Interactions	Other Interactions
Target protein: human Aβ_25-35_			
Phloroglucinol	−3.19	Gly25, Asn27, Ile32	Ala30 (Pi-Alkyl), Ser26 (Pi-Lone pair)
Eckol	−4.73	Gly25, Ser26, Asn27	Ile31 (Pi-Alkyl), Ala30 (Pi-sigma)
Dioxinodehydroeckol	−4.94	Ser26, Leu34	Ala30 (Pi-sigma, Pi-Alkyl), Ala30 (Pi-Alkyl), Ile32 (Pi-Alkyl)
Dieckol	−3.51	Gly25, Ile32, Ala30, Ser26, Gly29	Ile31 (Pi-sigma), Ile31 (Pi-Alkyl), Ile32 (Pi-Alkyl)
PFFA	−5.33	Gly29, Lys28, Asn27, Ile31, Leu34, Gly33	Ala30 (Pi-Alkyl)
Target protein: bovine insulin			
PFFA	−5.03	Ser12 (A), Gln15 (A), Glu17 (A), Asn18 (A), Asn3 (B), Phe1 (B)	Tyr14 (Pi-Amide stacked)
